# mTOR's role in ageing: protein synthesis or autophagy?

**DOI:** 10.18632/aging.100070

**Published:** 2009-07-20

**Authors:** Sarah L. Hands, Christopher G. Proud, Andreas Wyttenbach

**Affiliations:** ^1^ School of Biological Sciences, University of Southampton, Southampton, Boldrewood Campus, Basset Crescent East, SO16 7PX, UK; ^2^ School of Biological Sciences, Human Genetics Division, University of Southampton, Duthie Building, Southampton General Hospital, Southampton SO16 6YD, UK

**Keywords:** ageing, TOR, protein synthesis, autophagy, rapamycin

## Abstract

The molecular and cellular mechanisms that regulate ageing are currently under scrutiny because ageing is linked to many human diseases. The nutrient sensing TOR pathway is emerging as a key regulator of ageing. TOR signaling is complex affecting several crucial cellular functions and two such functions, which show clear effects on ageing, are protein synthesis and autophagy.  In this article we discuss the relative importance of both these processes in ageing, identify how TOR regulates translation and autophagy and speculate on links between the TOR signaling network and ageing pathways.

## Introduction

The mechanisms involved in ageing and longevity are currently receiving a great deal of attention, perhaps reflecting the impending socioeconomic impacts of a more ‘elderly' society. Recent research has revealed roles for the protein kinase termed ‘the target of rapamycin' (TOR) in modulating lifespan, and for two of the processes which TOR regulates, i.e. protein synthesis and autophagy. Studies in diverse model organisms have shown that impairment of TOR signaling leads to increased life span.

**Figure 1.**
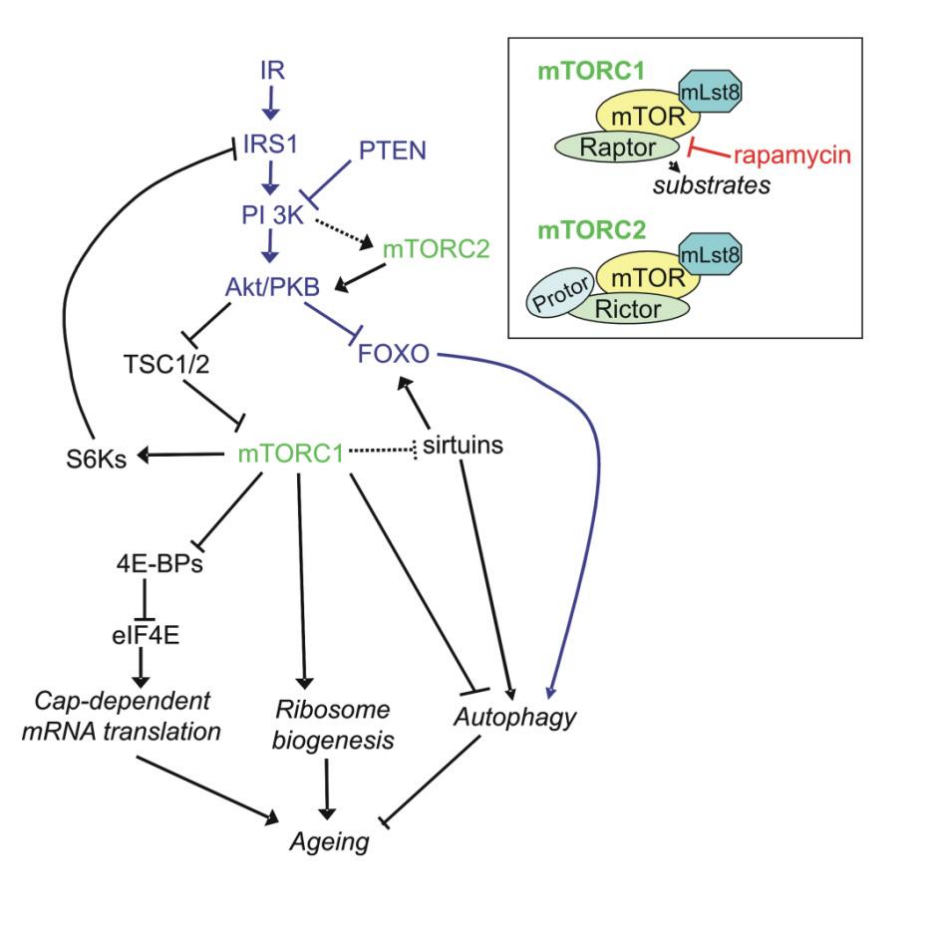
Signaling pathways linking mTORC1 and mTORC2 to ageing via protein synthesis and autophagy. It should be noted that many links have been made in *C. elegans *and *D. melanogaster* and the reader is referred to Table 1 for homologs of the mammalian proteins in this figure.  Inset: components of mTORC1 and mTORC2.

The mammalian target of rapamycin, mTOR, like its orthologs in other eukaryotes, is a multidomain protein kinase that interacts with other proteins to form two main types of complex, mTOR complexes 1 and 2 (mTORC1 and mTORC2) (inset Figure 1). Signaling through mTORC1 is much better understood than signaling through mTORC2: mTORC1 is an important node in cellular regulation impacting on cell growth that is linked to ageing [1]. Signaling through mTORC1 is activated by hormones, mitogens and growth factors, requires amino acids, and is negatively regulated by stressful conditions, such as decreased energy (ATP) availability. While both mTORC1 and mTORC2 contain mLST8 (also termed GβL), mTORC1 associates with raptor, which binds to proteins that are direct substrates for mTORC1 and mTORC2 binds to rictor. Although TOR's name reflects the fact that rapamycin can inhibit TOR function via an association of FKBP12 with rapamycin which binds to the FKBP12-Rapamycin Binding (FRB) domain of TOR, only (m)TORC1 is sensitive to this drug in the short-term, and not all functions of this complex are blocked by rapamycin. Dysregulation of mTORC1 signaling contributes to several human diseases - e.g., cancers, cardiac hypertrophy and tuberous sclerosis [2-4].  Indeed rapamycin, or its analogs (rapalogs), are in clinical use or trials for a number of diseases.

mTORC1 regulates a range of essential cellular functions, the best understood of these being protein synthesis (mRNA translation), which is positively regulated by mTORC1 (Figure 1) [5]. Conversely, mTORC1 signaling impairs autophagy [6], a degradative process through which proteins and other macromolecules are broken down. This article focuses on the fact that both protein synthesis and autophagy are implicated in regulating lifespan and ageing and investigates how mTORC1 links into the ageing signaling network.

### 1) Protein synthesis

Two widely-conserved effectors of mTORC1 which are linked to the translational machinery and its control are eukaryotic initiation factor (eIF) 4E and the kinases that phosphorylate S6, a component of the small (40S) ribosomal subunit (termed S6Ks). The physiological function of the phosphorylation of S6 [7] and the other S6K substrates functionally associated with mRNA translation requires clarification. However, the function of eIF4E is far better understood. eIF4E binds the cap structure found at the 5'-end of cytoplasmic mRNAs and also interacts with other proteins, in particular the multidomain scaffold protein eIF4G [8]. eIF4G, in turn, interacts with several other proteins. One of these is the poly(A)-binding protein (PABP). The interaction of eIF4G with eIF4E and PABP circularizes the mRNA (by bringing together its 5'- and 3'-ends), which markedly enhances its translation. eIF4G also, indirect-ly, recruits 40S subunits to the mRNA to initiate translation at the 5'-end. eIF4E and its interaction with eIF4G are therefore considered to be very important for the initiation of mRNA translation. The eIF4E:eIF4G interaction is blocked by the interaction of eIF4E with small phosphoproteins termed eIF4E-binding proteins, 4E-BPs. The best understood of these is mammalian 4E-BP1, which is directly phosphorylated by mTORC1: this leads to its release from eIF4E, allowing eIF4G and its partners to bind [8]. In this way, mTORC1 signaling positively regulates eIF4E activity.

**Table 1.** List of autophagy and protein synthesis homologs referred to in this review in S. cerivisiae, mammals, C. elegans and D. melanogaster and their effects on lifespan when known.

**Mammalian**	***S. cerivisiae***	***C. elegans***	***D. melanogaster***	**Function**	**Effect on longevity**
Unc-51 like kinases 1 & 2 (ULK1&2)	Atg1	Unc-51	DrATG1	Induction of autophagy	Loss of function mutation increases tissue ageing & decreases lifespan in *C. elegans* [49]
APG5	Atg5	Atgr-5	DrATG5	Autophagosome assembly	
Beclin-1	Atg6	Bec-1	DrATG6	Autophagosome assembly	Loss of function mutation increases tissue ageing & decreases longevity in *C. elegans* [49]
APG7	Atg7	Atgr-7	DrATG7	Autophagosome assembly	Knockdown/mutation reduces lifespan in *D. melanogaster* and *C. elegans *[49, 50]
LC3 (APG8)	Atg8	Igg-1	DrATG8	Autophagosome assembly	Reduced expression decreases longevity & enhanced expression increases lifespan in *D. melanogaster* [54,55]
APG12	Atg12	Igg-3	DrATG12	Autophagosome assembly	
WIPI1	Atg18	Atgr-18	CG11975	Recruitment of protein to vesicle membrane	Loss of function mutation increases tissue ageing and decreases lifespan in *C. elegans *[49]
mTOR	TOR	Let-363	DrTOR	Repression of autophagy	Inhibition extends lifespan in yeast, *C. elegans *and *D. melanogaster *[20-22]
S6K	Sch9p	Rsks-1	dS6K	Phosphorylates S6, a component of the 40S ribosomal subunit	Impairing expression in *C. elegans* & *D. melanogaster* extends lifespan [37, 39]
eIF4E	CDC33	Ife-1 - Ife-5	eIF4E-4	Translation initiation (binds mRNA's 5'-cap)	Knockout in *C. elegans* extends lifespan independent of Daf-2 & TOR pathways [39]
Mnk1 and Mnk2	-	Mnk-1	Lk6	eIF4E kinase	Affects lifespan in *D. melanogaster *[43]
FoxA 1, 2 & 3	PHA-4	Pha-4	forkhead	Transcription factor	Enhanced activity increases lifespan [71]
FOXO 1, 3 and 4	-	Daf-16	dFOXO	Transcription factor	Overexpresssion increases lifespan in *D. melanogaster* and *C. elegans *

Proper accuracy and control of protein synthesis is essential. For example, the accumulation of mistranslated and potentially misfolded proteins can lead to neurodegeneration [9]. Furthermore, protein synthesis is also a costly process, consuming both amino acids and energy. Indeed, the proportion of cellular energy used in protein synthesis is estimated to be as high as 30-40% of total ATP (and GTP) [10,11]. This consideration is important not only with respect to the overall cellular energy ‘economy' but also because the production of ATP in mitochondria is associated with the generation of reactive oxygen species (ROS) which may have damaging effects on cellular components. Interestingly, mTORC1 signaling plays a role in regulating mitochondrial function [12-14]. A number of studies in a broad range of organisms have now demonstrated links between mTOR, protein synthesis and ageing/life span. The overall thrust of these studies is that inhibiting protein synthesis or (m)TOR signaling can extend life span.

### 2) Autophagy

Autophagy is a second key process that is regulated by mTORC1 (Figure 1). Autophagy is a process by which cargo, such as long-lived proteins and cytoplasmic organelles, is sequestered and delivered to the lysosomes. Based on the cellular mechanisms of cargo delivery to lysosomes, three different types of autophagy have been described in mammalian cells [15]. Chaperone-mediated autophagy (CMA) is a form of autophagy wherein a soluble pool of cytosolic proteins is targeted to lysosomes for selective degradation [16]. Cytosolic proteins with a CMA-targeting motif bind to a receptor protein, the lysosomal-associated membrane protein (LAMP-2A), are translocated across the membrane and are degraded within the hydrolase-rich lumen. In addition to CMA, there are two other types of autophagy, macro-and microautophagy, which involve mainly non-selective engulfment of cytosolic regions, including organelles and soluble proteins. Macroautophagy is the most extensively characterized process where de novo-formed limiting membranes sequester regions of the cytosol ("bulk degradation"), but also selectively sequestrate cellular organelles and protein aggregates into autophagosomes. Such double-membrane vesicles (autophagosomes) then acquire proteases that are responsible for degrading engulfed material via a fusion event with endosomes and lysosomes. The formation and fusion of the autophagic compartment with lysosomes is regulated by a protein-to-protein and a protein-to-lipid conjugation controlled by the beclin-VPS34 (vacuolar protein sorting) and the mTORC1 intracellular kinase complex.

Although the process of autophagy is still poorly characterized (at least in mammalian cells), much progress has been made since yeast genetic studies identified the first autophagy-related genes (ATGs) about 10 years ago. Now, more than 30 such genes have been discovered. Most of these are conserved throughout eukaryotes. It is well established that normal cellular function relies on surveillance mechanisms, molecular chaperones and proteolytic systems and that many of these functions decline with age. It is evident that the build-up of damaged cellular components in ageing cells and tissues is, at least partly, attributable to a decline in autophagy, including CMA [17,18].  This has been proposed to be due to a decrease in the clearance of vacuoles, for example the accumulation of lipofuscin with age in the lysosome could diminish lysosomal function or the lysosome could be damaged by toxic protein products [19]. A decrease in the sensitivity of autophagy to regulation by insulin and glucagon has also been suggested to play a role in modulation of autophagy with age [20,21].

There is extensive evidence that macroautophagy (referred to as autophagy from here on) is negatively regulated by mTORC1 (eg [22] and references therein). However, the mechanisms by which mTORC1 controls autophagy are not well established. The mechanisms regulating the initial stages of autophagy, at least, appear to involve mTOR-dependent phosphorylation of ULK1 (UNK-51-like kinase), a mammalian serine/threonine protein kinase (Atg1 in yeast) which forms a 3-MDa complex with Atg13 and FIP200 [23-26]. It has also been suggested that mTOR acts on ATG genes through the regulation of the phosphatase PP2A [27]. Less direct ways by which mTOR may regulate autophagy are via S6K and its transcriptional targets or signaling through Akt (Figure 1).  For example, using RNAi knockdown in cell lines, S6K has recently been shown to be required for the starvation-induced autophagic response [28].

In this review we concentrate on TOR dependent regulation of autophagy, but it should be noted that autophagy is also regulated in a TOR independent manner, either via IP3 [29] or via other protein kinases. For example, cAMP-dependent protein kinase (PKA) was shown to inhibit the induction of autophagy in yeast [30] and Atg1, 13 and 18, which are required for autophagy, are PKA substrates [31]. Furthermore, the AMP-activated protein kinase (AMPK), an important energy sensor, regulates the phosphorylation of the cell-cycle regulator, p27.  p27 can maintain autophagy in a human cell line [32] and its regulation is proposed to be an important determinant of whether a cell enters a survival pathway (via autophagy) or apoptosis. All these effectors of autophagy, including TOR and ATG genes, have been linked with longevity. For a recent in-depth review on the molecular mechanisms by which autophagy genes interact with longevity pathways in diverse organisms the reader is also referred to the paper by Vellai [33].

### 3) TOR-dependent regulation of ageing via protein synthesis and autophagy

Recently, it has been proposed that the main driver of ageing is TOR signaling rather than ROS [34]. Inhibition of the TOR pathway extends lifespan in yeast, worms and flies [35-37] and a recent, notable study showed that rapamycin, an inhibitor of mTOR, fed late in life extends lifespan in genetically heterogeneous mice [38]. Below, we discuss the ways in which protein synthesis and autophagy may contribute to the regulation of lifespan by TOR. We summarize drug studies in cells and animals as well as evidence obtained by genetic analyses in various model organisms. For information on gene homologues and their effects on longevity across model organisms the reader is referred to Table 1.

### 3.1) mTOR, translation, and life-span

In the nematode worm *C. elegans*, several studies have shown that decreasing the amount of proteins involved in mRNA translation (ribosomal proteins, initiation factors) extends life span - examples of the latter include eIF4E and eIF4G as well as eIF2 and eIF2B. Both eIF4E and eIF4G can be controlled by mTORC1 [39,40]. Reduced expression of TOR or S6 kinase also led to longer life. It has been argued that decreased protein synthesis rates might extend lifespan by reducing energy consumption and thus diminishing respiration and ROS production. However, reducing translation still increased lifespan in animals with decreased respiration, suggesting that protein synthesis affects life span independently of effects on energy usage or oxygen consumption. Other data indicate that the effects of decreased mTORC1 signaling may be mediated through reduced mitochondrial oxidative metabolism (e.g. [41] and discussion therein). There appears to be a complex interplay between mitochondria and (m)TOR, with signaling proceeding in both directions between them [13]. mTORC1 signaling promotes the transcription of genes involved in mitochondrial function [12] likely as part of a programme of events to generate the energy required by anabolic processes such as protein synthesis. Interestingly, the effect of yeast TORC1 on mitochondrial function to influence chronological life span (CLS, the period in which yeast cells remain viable in a non-dividing state) occurs not via mitochondrial bio-genesis, but primarily through translational regulation of OXPHOS complexes [14].

The role of eIF4E in longevity has been the subject of several recent studies.* C. elegans* possesses several isoforms of eIF4E, with different patterns of expression and differing specificities for the different 5'-cap structures found in this organism. The product of the* ife-2* gene is widely expressed in somatic tissues and binds to both the 7-monomethyl and 2,2,7-trimethyl guanosine cap structures found in mRNAs in this species. Knockout of this gene has been shown to extend lifespan, in a manner distinct from the DAF-16 (FOXO) pathway (Figure 1) [39,42], and enhances resistance to oxidative stress, UV irradiation and heat shock, as well as to starvation. It should be noted that in the study of Hansen and colleagues [39] an *ife-2* mutant did not extend lifespan in a daf-16 mutant background, implying that the extension of lifespan due to loss of *ife-2* may be DAF-16 dependent. Interestingly, both increasing and decreasing the levels of the eIF4E kinase, Lk6, increases lifespan in *Drosophila* [43].

It remains to be established whether manipulations that inhibit general protein synthesis affect longevity due to the decreased rates of overall protein synthesis or perhaps to the concomitant upregulation of the translation of specific mRNAs whose products help confer resistance to stress. A combination of these effects may, of course, be involved. The effects of manipulating eIF4E activity may be especially relevant here, since eIF4E is involved in cap-dependent translation while many mRNAs for ‘stress' proteins are encoded by mRNAs by mechanisms that either show a low requirement for the cap-dependent translation machinery or are translated by cap-independent mechanisms such as internal ribosome entry segments (IRESs) [44]. Since longevity is promoted by decreasing the levels of initiation factors required for general translation (eIF2β[39] or eIF2Bδ[40]) or of ribosomal proteins [39], it seems that impairing general protein synthesis, rather than specifically inhibiting cap-dependent translation, extends lifespan.

The regulation of eIF4E's activity by 4E-BPs (TOR substrates) could provide a mechanism by which TOR controls longevity. However, no structural ortholog of the 4E-BPs has been identified in *C. elegans* although it is possible that functional orthologs do exist. However, knocking down TOR extends lifespan further in *ife-2* deleted worms, indicating that the effects of TOR and *ife-2* are mediated through distinct mechanisms [42], further discussed in section 4.2. Thus, TOR's effects on lifespan in the worm model appear to operate by additional mechanisms. For example, TOR signaling promotes ribosome biogenesis, a costly process that is needed for cell growth and division, across the eukaryota from yeast to mammals [45]. Since the number of ribosomes in a cell governs the cellular capacity for protein synthesis, decreased ribosome production may be involved in extending lifespan in animals where TOR signaling is downregulated.

### 3.2) Autophagy and the modulation of life-span

As mentioned above, inactivation of TORC1 via treating cells with rapamycin or nitrogen starvation induces autophagy [46,47].  The extension of lifespan due to TOR inhibition could potentially occur solely via TOR's effects on protein synthesis (see above).  However, recent studies in *C. elegans* suggest a direct role for autophagy in modulating longevity, as inactivation of autophagy genes (*bec-1*, *unc-51*, *atg-18*) specifically prevents inhibition of TOR activity from extending lifespan [48,49]. This indicates that TOR and autophagy act via the same signaling pathways to affect lifespan.

A recent study using loss of function mutational analysis in *C. elegans *showed a clear acceleration in tissue ageing and a reduced lifespan in worms with loss of function mutations in the autophagy genes, *bec-1*, *unc-51* and *atg-18* [49]. This shows the importance of autophagy in regulating normal lifespan. These findings support an earlier study using RNAi knockdown of *atg-7* which reduced lifespan of wild-type *C. elegans *[50]. Some other experiments in *C. elegans* using RNAi knockdown of *atg-7* or *bec-1*, however, showed no significant effects on lifespan [48,50,51]. These negative findings could be explained by the finding that residual Atg activity can still produce significant autophagic flux (e.g. in a mammalian cell system) [52,53].  In *Drosophila*, mutation of *Atg7* and reduced expression of *Atg8* each decreased longevity [49,54] and upregulation of the autophagy gene *Atg8* increased longevity [55]. Also in yeast, autophagy has been implicated in ageing (reviewed in [33]). For example, a recent study showed that the CLS in yeast is reduced by deletion of *Atg1* or *Atg7*, both required for autophagy in yeast [56]. Therefore, loss of autophagy controlled by TOR accelerates ageing. This is not unexpected because of the importance of autophagy in maintaining a healthy cellular environment where damaged proteins and organelles can be eliminated.

### 4) The role of mTOR in ageing controlled by dietary restriction and insulin signaling

### 4.1) Insulin signaling

The effect of TOR on lifespan operates downstream of the insulin signaling pathway (Figure 1), as indicated by the observation that the increase in lifespan in an insulin signaling mutant cannot be further extended by mutations in components of the TOR pathway [21]. A link between insulin signaling, ageing and autophagy was initially described when studies in rodent liver showed that an increase in insulin with age causes an inhibition of autophagy and that the ability of glucagon to upregulate autophagy is reduced with increasing age [57,58].  More recently, FOXO (Daf-16) has been shown to directly control the transcription of autophagy genes, including members of the Atg8 family (*LC3, Gabarapl1*) and regulators of autophagy, *Bnip3*, and *Atg12l *[59,60]. Upregulation of FOXO also induces autophagy in *Drosophila* [61], *C. elegans* [59] and mouse muscle fibres [60]. In addition, a mutation of FOXO caused a reduction of starvation-induced autophagy in the fat body of *Drosophila* [61]. The *C. elegans* study showed that the upregulation of autophagy in skeletal muscle via Daf-16 was independent of mTOR, as demonstrated by inhibition of mTOR by rapamycin or knockdown [59].  Knockdown of a component of mTORC2 (rictor) did, however, result in FOXO-mediated induction of autophagy. The authors explain the apparent discrepancy between the lack of effect of mTOR inhibition and the positive effect of mTORC2 inhibition on autophagy by a negative feedback of S6K on Akt/PKB activity.  It has indeed become apparent that Akt signaling can be both positively and negatively regulated by mTOR, depending on the TOR complex. As described above, S6K is a target of mTORC1. S6K phosphorylates IRS1 at inhibitory sites, inhibiting activation of Akt [62,63], upregulating autophagy.  On the other hand, mTORC2 has been shown to phosphorylate S473 of Akt, hence activating Akt and downregulating autophagy [64].  This is consistent with observations in the *Drosophila *fat body, whereby signaling through TOR and PI3K is necessary and sufficient to suppress starvation-induced autophagy and yet S6K promotes autophagy [65].  The balance between mTORC1 and mTORC2 signaling therefore could be critical in the regulation of Akt and hence autophagy and ageing.

Further evidence linking insulin signaling with autophagy comes from a mouse with targeted deletion of PTEN, in the liver. PTEN is a lipid phosphatase that reduces PI3K activity and hence an antagonist of insulin signaling, whose elimination will result in activated Akt and thus mTORC1.  Autophagic degradation in the liver of this mouse was significantly reduced [66]. In *C. elegans*, downregulation of the autophagy gene Bec1 inhibited the longevity phenotype of the Daf-2 insulin receptor mutant [67], indicating that the extension of lifespan due to alterations in insulin signaling may occur, at least in part, via autophagy.

### 4.2) Dietary restriction and sirtuins

Another well-established mechanism for promoting longevity is dietary restriction. Dietary restriction in many organisms, including *Drosophila*,* C. elegans* and rodents, is known to induce autophagy [65,68,69], which is to be expected given that TOR signaling is impaired when amino acids levels are low.  On the other hand, it has been demonstrated that the decline in autophagy with increasing age can be prevented by caloric restriction in mice [70].  Two autophagy genes, *bec-1* and *atg-7*, have been shown to be required for the longevity phenotype of the inherent dietary restriction *C. elegans *mutant eat-2 [48,51] indicating that autophagy is required for the lifespan extension induced by dietary restriction.

The FoxA family of transcription factors is involved in multiple physiological processes including the regulation of longevity in response to dietary limitation and related manipulations [71]. Sheaffer and colleagues identified the AAA+ ATPase *ruvb-1* as a component of the TOR signaling pathway and as a negative regulator of the FoxA homolog* pha-4* in *C. elegans*. They showed that the effects on lifespan of inactivating TOR or the S6K homolog *rsks-1* requires *pha-4, *whereas the lifespan-promoting effect of mutations in eIF4E/*ife-2* does not require *pha-4*. This suggests that eIF4E and TOR affect longevity via distinct mechanisms. Therefore, nutrient availability may control longevity by affecting TOR signaling and repressing or enhancing *pha-4*/FoxA function.

The activation of autophagy due to dietary restriction in *C. elegans *was also shown to require PHA-4 (FoxA) activity [48]. *Pha-4* is required for the induction of increased numbers of autophagic vesicles under certain conditions [72], suggesting that changes in gene expression are required for this process (since *pha-4* is a transcription factor). In addition to repressing *pha-4*, TOR and *ruvb-1* regulate the nucleolar accumulation of components, termed box C/D snoRNPs that are involved in the maturation of rRNAs (which are made in the nucleolus). Impairing TOR/*ruvb-1* signaling is thus expected to interfere with ribosome production, likely explaining the decreased rates of protein synthesis seen in worms where TOR signaling or box C/D snoRNP function is perturbed [71]. It remains to be elucidated which functions of *pha-4* are involved in regulating lifespan.

Data from yeast point to a role for Sch9p, the probable yeast ortholog of S6K (and which is downstream of TORC1) in lifespan extension due to dietary restriction [73]. Impairing S6K/Sch9p activity (using an inactive ‘dominant negative' mutant) also extends lifespan in *Drosophila* [37]. Interfering with S6K expression in *C. elegans* also extended lifespan [39]. S6K has been implicated in ribosome biogenesis, in oxidative phosphorylation [14], in the regulation of nucleolar rDNA transcription [45] and in the control of the translation of mRNAs for ribosome proteins (although recent work has revealed that S6Ks are dispensable for the latter (reviewed in [7]). It therefore remains to be established how S6K orthologs affect lifespan.

Studies in yeast implicate TOR-mediated regulation of proteins called sirtuins in the control of replicative lifespan. Sirtuins are NAD^+^-dependent deacetylases that stabilize the rDNA locus and, interestingly, are also involved in the control of lifespan by caloric restriction, not only in yeast, but also in *C. elegans* and in* Drosophila *[74,75]. TOR may impair sirtuin activity through the induction of the Pnc1p nicotinamidase via the transcription factors Msn2p and Msn4p, whose nuclear localization is inhibited by TORC1 signaling [74]. Recently a role for Sirt1 (Sir2 in yeast) in upregulating autophagy was revealed [76].  In this study Sirt1 was upregulated in mice subjected to starvation and was necessary for the induction of starvation-induced autophagy.  Sirt1^-/- ^mouse fibroblasts were unable to stimulate basal rates of autophagy and Sirt1 interacted with Atg5, 7 and 8.  In yeast, TOR inhibition has been shown to extend lifespan by increasing Sir2 activity, the same mechanism thought to be involved in extending lifespan in response to caloric restriction [74].  Recent work in *Drosophila *shows that dSir2 interacts with and deacetylates p53, which mediates, at least in part, the lifespan extending effects of dietary restriction [77]. Therefore, sirtuins provide an important link between dietary restriction, TOR signaling and ageing, a link which may arise due to the regulation of autophagy by sirtuins, via deacetylation and hence activation of FOXO [75] (see signaling diagram) and/or by direct deacetylation of autophagy components [76]. Given that sirtuins control processes that defend cells against oxidative stress which, in turn, can be regulated by TOR (see below), it is noteworthy that SIRT3 physically interacts with the daf-16 homolog FOXO3 within mitochondria [78], organelles that are signal integrators of oxidative metabolism and perhaps ageing.

### 5) The interplay of TOR, oxidative/mitochondrial metabolism and ageing

The relationships between TOR signaling and oxidative and/or mitochondrial metabolism are complex. While ROS or peroxides induced by growth factors or UV activate mTOR (e.g. [79-83]), exogenous hydrogen peroxide inhibits mTOR (>100 μM) [84]. mTOR itself has been shown to both increase [85] and decrease [86] ROS levels. However, these studies were performed on transformed cell lines or LPS-stimulated hepatocytes and it remains to be seen whether the modulation of mTOR under basal conditions in primary tissues causes significant changes in cellular redox-homeostasis.

There is increasing evidence that mTOR-regulated autophagy plays a dual role in the cellular response to oxidative stress. On the one hand autophagic pathways are compromised due to ageing and in age-related disorders such as Alzheimer's and Huntington's Disease, which could lead to accumulation of oxidized proteins in aged cells under normal growth conditions [87,88]. This suggests that upregulation of autophagy protects against free radical damage. Indeed, pharmacological induction of autophagy decreased the age-dependent accumulation of oxidatively-damaged mitochondrial DNA in rat liver [89,90].  This view is also supported by genetic studies: for example, enhanced Atg-8 expression in old *Drosophila* brains extends adult life span, promotes resistance to oxidative stress and reduces the accumulation of oxidized proteins [55]. Additionally, the age-dependent reduction in autophagy may cause the build-up of severely damaged mitochondria, further increasing oxidative stress and causing additional molecular, cellular and tissue damage with age. Hence autophagy may provide the front line of defense against oxidative stress. The recent finding that Atg4, an essential protease that controls the lipid modification of Atg8 and autophagosome formation, is a direct target for oxidation by hydrogen peroxide, further underlines this idea. It has also been suggested that low levels of ROS provide a signal to regulate autophagic survival and death processes (reviewed in [91]). Collectively, the evidence suggests that free radicals are upstream and downstream of mTOR and numerous feedback and feed forward loops exist. For a conceptual view on the role of ROS versus TOR in ageing the reader is referred to the recent review by Blagosklonny [34].

On the other hand, in addition to its role in promoting cell survival and increasing life span, autophagy can actually result in cell death (autophagic or type II cell death) [92] that is observed under conditions of oxidative stress. It has been demonstrated that hydrogen peroxide induces autophagy via a novel autophagy signaling mechanism that links PARP-1 activation to the LKB-1-AMPK-mTOR pathway. Hence PARP-1 activation appears to promote autophagy. Poly(ADP-ribose) polymerases are well known for repairing single and double DNA strand breaks and therefore they play a role in maintaining genomic stability, preventing carcinogenesis and ageing (reviewed in [93]). In this context it is important to note that p53, the "guardian of the cellular genome" that senses cellular damage, both positively [94] and negatively [95] regulates autophagy. While nuclear p53 transactivates autophagy-enhancing genes [94,96], the cytoplasmic pool of p53 can act as a negative regulator of autophagy. Knockout of p53 stimulates autophagy in human, mouse and *C. elegans* cells [95]. The tumor suppressor p53 favors organismal ageing [97,98]. p53 gain-of-function mutations are linked to accelerated ageing and premature death in mice [99] and humans [100], whereas a dominant negative p53 transgene increases longevity in Drosophila [101] and loss of function mutations of the *C. elegans* p53 ortholog *cep1* extends life span [102]. Hence, a recent study tested whether a mutation in *cep1* increases life span in worms via an increase in baseline autophagy. Tavernarakis and colleagues found that RNAi against the autophagy gene *bec-1* significantly reduced life span extension caused by cep-1 mutants [103]. It is likely that this functional link between increased life span and increased autophagy due to the *cep-1* mutation are TOR dependent as in mammalian cells knockout and knockdown of p53 causes an inhibition of mTOR [95]. The exact molecular mechanisms and signaling between p53 and (m)TOR activity and its relationship to ageing is expected to be rather complex and remains to be determined.

## Conclusion

There is overwhelming evidence that cellular mechanisms and signaling pathways regulating ageing are controlled by mTOR. Here we have highlighted important studies that support a role for both mTOR dependent protein synthesis and autophagy in ageing. Genetic approaches show a clear overlap between signaling networks that control ageing and autophagy. An increase in autophagy controlled by mTOR extends life span via dietary restriction and insulin signaling. Causal links, if any, between mTOR dependent ageing and the regulation of cellular redox-homeostasis are less clear and are only beginning to emerge. Key factors that regulate longevity and enhance autophagy such as p53, FOXOs and sirtuins are therefore targets in the fight against age-related diseases. Several genetic studies provide good evidence that decreasing protein translation extends life span (e.g. via elF4E). While there are no established links between translation, insulin signaling and longevity, dietary restriction appears to affect longevity via S6Ks, perhaps also by acting on ribosome biogenesis. However, more work is now needed to establish the molecular links for mTOR's role in ageing via protein synthesis. Such future work will establish whether and how to harness the protein synthesis machinery for therapeutic purpose.
